# Do *Candida albicans* Isolates with Borderline Resistant Micafungin MICs Always Harbor *FKS1* Hot Spot Mutations?

**DOI:** 10.3390/jof7020093

**Published:** 2021-01-28

**Authors:** Kathrin Spettel, Sonia Galazka, Richard Kriz, Iris Camp, Birgit Willinger

**Affiliations:** 1 Division of Clinical Microbiology, Department of Laboratory Medicine, Medical University of Vienna, 1090 Vienna, Austria; kathrin.spettel@meduniwien.ac.at (K.S.); sonia_galazka@hotmail.com (S.G.); iris.camp@meduniwien.ac.at (I.C.); 2 Division of Infectious Diseases and Tropical Medicine, Department of Medicine I, Medical University of Vienna, 1090 Vienna, Austria; richard.kriz@meduniwien.ac.at

**Keywords:** *FKS1* mutations, echinocandin resistance, *Candida albicans*, next-generation sequencing, micafungin

## Abstract

Antifungal susceptibility testing is important in guiding patient therapy due to an increasing number of resistant *Candida* isolates. In the clinical strain collection of the Austrian resistance report (AURES), a high number of micafungin-resistant *C. albicans* isolates (18.2% 49/269) was detected in seven different centres in Austria from 2011–2016. Most of these isolates showed a micafungin MIC value that was just above the clinical breakpoint (CB) established by EUCAST (0.016 mg/L). The aim of this study was to analyse whether *C. albicans* strains showing a micafungin MIC value of 1–2 dilutions above the CB (0.032 mg/L and 0.064 mg/L) are associated with mutations in *FKS1* hotspot (HS) regions. 115 *C. albicans* candidemia strains showing a micafungin MIC one or two dilutions above the EUCAST CB (0.032 mg/L and 0.064 mg/L) were categorized as borderline resistant and screened for mutations in *FKS1* HS1, HS2, and HS3 regions, which are known locations for the development of echinocandin resistance. For this purpose, we implemented targeted resequencing utilizing a next generation sequencing technology. No missense mutations could be detected in *FKS1* HS1, HS2, and HS3 in any of the 115 isolates, which indicated that resistance conferred by alteration of *FKS1* seems unlikely.

## 1. Introduction

Nosocomial infections, such as invasive candidiasis, are associated with significant case fatality rates, especially in immunocompromised patients. Currently, blood stream infections that are caused by *Candida* spp. are among the most common infections. One of the reasons for this development is the increase in the number of patients at risk, such as transplant recipients as well as cancer and immunosuppressed patients. Santolaya et al. gathered data regarding the most frequently occurring species in candidemia belonging to ICU patients of 23 hospitals in eight countries. *C. albicans* was responsible for most cases of candidemia, accounting for 43.8% of all cases [1]. Bassetti et al. observed 7.07 candidemia cases per 1000 ICU admissions with a 30-day mortality rate of 42% in 23 European countries [2]. Echinocandins are first-line agents for the treatment of severe systemic candidiasis, as suggested by the Infectious Diseases Society of America (IDSA) as well as the European Society of Clinical Microbiology and Infectious Diseases (ESCMID) [3,4].

Echinocandin resistance has been associated with intrinsic or acquired mutations in certain genetic regions that encode for the subunits of the β-(1,3)-D-glucan synthase (Fks), which is responsible for the synthesis of β-(1,3)-D-glucan, an essential polysaccharide component of the fungal cell wall. Mutations in these regions could lead to a reduced binding affinity and, thus, induce echinocandin resistance. Point mutations causing echinocandin resistance have been almost exclusively observed in three conserved regions. These regions are also known as “hotspot” regions (HS), which represent the binding domains of the echinocandins [5]. In *C. albicans,* these specific point mutations have been detected at the amino acid positions 641 to 649, which represent the HS1 region, and 1345 to 1365, which represent the HS2 region [6]. Furthermore, a third hot spot region, which is located between HS1 and HS2 at residues 690 to 700, was associated with the acquisition of echinocandin resistance [7].

In Austria, concerns were raised when an increasing number of *C. albicans* strains from candidemic patients showed resistance to micafungin. These strains were described by the annually published Austrian resistance report (AURES). Since 2007, *Candida* isolates from candidemic patients are collected and analysed for this report. Starting with 2014, an increase of micafungin resistance was described, culminating in 2017 with 26.8% (22/82). All of the isolates except a single one were only one or two dilutions above the breakpoint [8,9,10,11].

Because all of strains published by AURES are collected at our department we retested all strains in order to confirm the published resistance data [12]. The MICs of fluconazole, voriconazole, posaconazole, itraconazole, isavuconazole, anidulafungin, caspofungin, and micafungin of all isolates were determined while using the microdilution reference method as described by EUCAST. Out of 754 *C. albicans* isolates that were collected from seven different Austrian centres 16% (122/754) were categorized as resistant to micafungin. In 115 isolates, the MICs to micafungin were either 0.032 mg/L or 0.064 mg/L and, thus, only one to two dilutions above the previous clinical breakpoint (CB).

In a previous study, we were able to show that *C. albicans* and *C. glabrata* with minimal inhibitory concentrations (MIC) of more than two dilutions above the clinical breakpoints are associated with missense mutation in the hot spot regions in *FKS* genes [13]. Therefore, our aim was to investigate whether isolates with a MIC of micafungin just above the CB show a resistant genotype with *FKS1* hotspot mutations. Thus, we examined whether these isolates that are classified as borderline resistant to micafungin lack target mutations in the hotspot regions associated with echinocandin resistance.

## 2. Materials and Methods

### 2.1. Sampling

**Figure 1:**
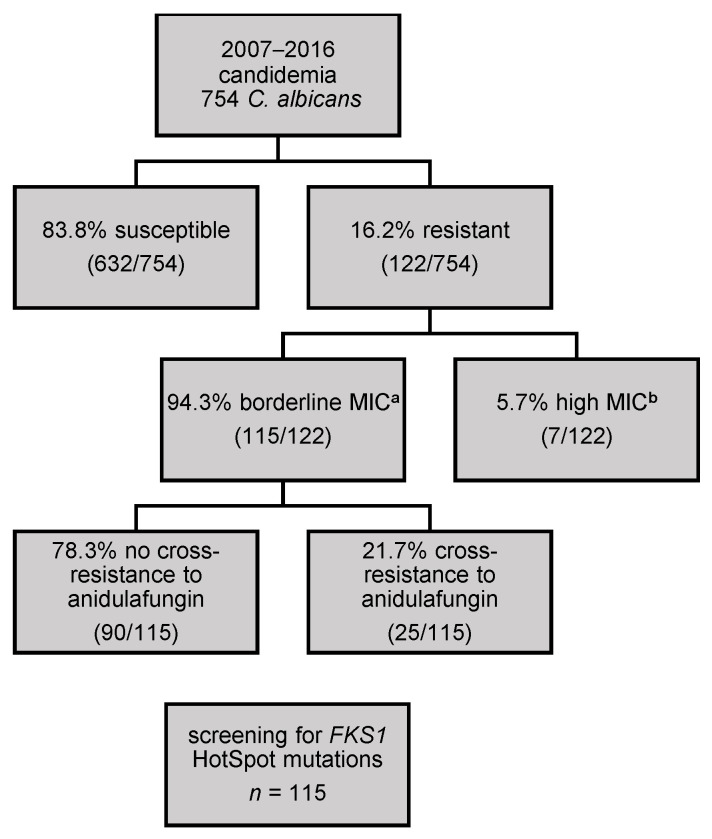
The selection of the *C. albicans* strains to be sequenced. **^a^** borderline micafungin MIC: are classified as one or two titres above the clinical breakpoint of EUCAST (0.032 mg/L or 0.064 mg/L) **^b^** high micafungin MIC: three titres or more above the EUCAST clinical breakpoint (≥0.125 mg/L).

115 *C. albicans* isolates showing micafungin MIC values of one (109/115) or two dilutions (6/115) above the former EUCAST CB and already tested and analysed, as previously described [12], were selected to screen for *FKS1* hot spot mutations. These strains had been collected from 2007–2016 and classified as borderline resistant to micafungin (see Figure 1).

### 2.2. DNA Extraction

These 115 cryopreserved isolates were thawed, plated on Sabouraud dextrose agar (SDA), and then incubated at 37 °C for 24 h. For DNA extraction, we used a modified SDS CTAB chloroform based method [13,14]. Fungal material from the pure culture was mixed with Tris-EDTA-buffer and glass beads, followed by the addition of sodium dodecyl sulphate (SDS) and proteinase K. The mixture was then incubated for 30 min at 65 °C and then vortexed while using the Homogenizer FastPrep (MP Biomedicals, Santa Ana, CA, USA). Sodium chloride was added, followed by the detergent hexadecyltrimethylammoniumbromide (CTAB), in order to increase the salt concentration. After the incubation, chloroform:isoamyl- alcohol 24:1 was added to the samples. The aqueous phase was transferred into a new tube and combined with 5 M ammonium acetate. Subsequently, isopropanol was added for DNA precipitation. This mixture was incubated at −20 °C overnight. Afterwards, the tubes were centrifuged, the supernatants were decanted, and the pellets were washed twice with ethanol 70%. The air-dried pellets were then resuspended in 50 µL Tris-Buffer. The Qubit dsDNA BR kit (Thermo-Fisher Scientific, Waltham, MA, USA) was used to quantify the DNA. In order to check the quality and purity of DNA, the 260/280 and 260/230 ratios were determined while using Nanodrop 2200c.

### 2.3. Next-Generation Sequencing and Library Preparation

Sequencing was carried out using a targeted resequencing approach on the Illumina MiSeq^®^ platform (Illumina Inc., San Diego, CA, USA). The amplicon sequencing was based on the 16S protocol that was described by Illumina [15]. To amplify the *FKS1* Hotspot 1, 3, and 2 regions, the KAPA Hifi Hot Start Ready Mix^®^ (Kapa Biosystems, Wilmington, MA, USA), which contains a high-fidelity proof-reading polymerase, was used. 12.5 ng extracted DNA were added to the mix and the PCR was carried out according to manufacturer’s instructions with an annealing temperature of 55 °C. Table 1 shows the sequences of the used primers [13,16]. The hotspot 1, 3 and 2 regions of one sample were pooled and purified using AMPure^®^ Beads (Beckman Coulter, Brea, CA, USA). In order to identify the samples after pooling of the library, the index PCR was carried out according to Illumina 16S protocol. After the index PCR, a second washing step using AMPure^®^ Beads was done to remove PCR components. Afterwards, the samples were diluted to 8 pM and then denatured according to Illumina 16 s protocol. 25% of PhiX control was added in order to receive an adequate diversity of the sequencing library. The sequencing run was carried out using a V2 Nano Flow Cell 2 × 250 ^®^ (Illumina, San Diego, CA, USA).

### 2.4. Bioinformatic Analysis

The quality of the NGS run was evaluated using FASTQC 0.11.4 [17]. Trimmomatic software 0.35 was used for the trimming of the primer sequences and filtering of low quality base calls [18]. The reads were mapped to the sequence of the *C. albicans* reference strain SC5314 using Bowtie2 2.2.7 [19]. SAM-Tools 0.1.19 was utilized for alignment. VarScan v2.3.9 was used as a variant caller for the detection of nucleotide substitutions, insertions, and deletions [20]. In the last step, SnpEff 4.270 was applied in order to predict the coding effects of the variants.

## 3. Results

### 3.1. Validation of the Sequencing Run

In order to validate the sequencing process, control strain SC5314 was sequenced and no discrepancies to the published sequences were found. The quality score Q30 in this run was 89.6%. The total number of reads was 1,482,998 and the yield was 363.36 Mbp. The total fraction of passing filter reads that were assigned to an index was 95.34%.

### 3.2. Sequencing Results of FKS1

**Figure 2:**
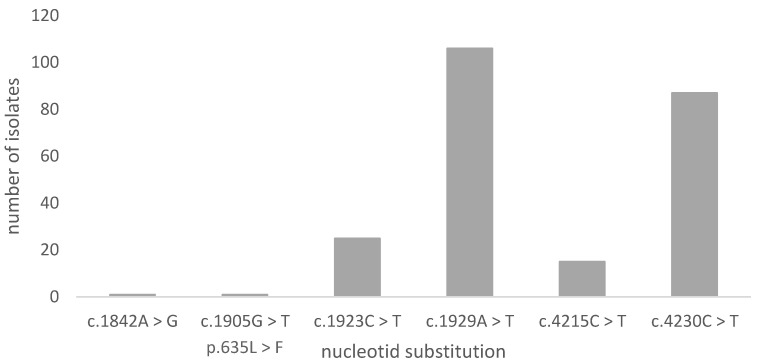
Variants in the *C. albicans* isolates in ascending nucleotide position. Altogether, 235 nucleotide substitutions were distinguished.

**Figure 3:**
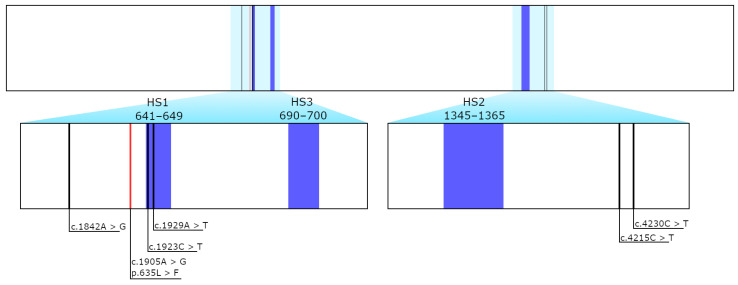
Sequenced hot spot regions in *FKS1* gene and the position of the detected variants.

Out of the 115 sequenced samples, no mutations were found in hotspot regions, with the exception of one sample showing a low frequency missense mutation (c.1905G > T/p.635L > F, frequency: 26.18%), which was located outside the hot spot region. The frequency suggests that this mutation is neither homozygous (~100%) nor heterozygous (~50%), but a subpopulation. Moreover, the position of this missense mutation is beyond the highly conserved regions of HS1, HS2, or HS3, and, thus, a direct influence on echinocandin susceptibility is unlikely. The mutations that are shown in Figure 2 and Figure 3 consist of five silent variants (c.1842A > G, c.4215CT, c.1923C > T, c.4230C > T, and c.1929A > T) and the mentioned low frequency missense mutation (c.1905G > T/L635F).

## 4. Discussion

In general, most of the *Candida* species exhibit good in vitro susceptibility to antifungal agents. However, the resistance to echinocandins in *Candida* spp. has appeared in recent years being the most common in *C. glabrata.* Additionally, breakthrough infections that are caused by *C. glabrata* have been reported in patients on micafungin therapy. In Austria, in recent years a high resistance rate for micafungin in *C. albicans* isolated from blood stream infections was observed, as described by Willinger in the Austrian Resistance Reports (AURES) [8,9,10,11]. In 2014, 23% (6/26) of *C. albicans* were described as resistant to micafungin. However, five of those strains (5/26) were only one-fold dilution above the EUCAST CB. Furthermore, it was shown that resistance to anidulafungin was considerably lower (2.1%). In 2015 and 2016, only 9.1% (2/22) and 10.3% (4/39) of the tested *C. albicans* were resistant, with all of them being only one- to two-fold dilutions above the EUCAST CB [9,10]. EUCAST collected MIC data for micafungin of 1569 *C. albicans* isolates. Of these, 156 (10%) isolates had a MIC of 0.032 mg/L or higher and, thus, were classified as being resistant by the EUCAST CB of 2018. One-hundred-fifty-three of these isolates were borderline resistant, i.e., they had a MIC of 0.032 mg/L or 0.064 mg/L. The EUCAST CB of micafungin seems to dissect the MIC distribution, raising the question as to whether these borderline resistant isolates still belong to the wild type distribution. The distribution of *C. glabrata* is similar, but the EUCAST CB is set one dilution higher at ≥0.032 mg/L. This leads to a considerably lower number of phenotypically resistant *C. glabrata* strains. In a previous study, we assessed the antifungal susceptibility in 1360 clinical *Candida* isolates that were collected from Austrian hospitals between 2007 and 2016 and detected a higher micafungin resistance rate in *C. albicans* (16.2%) than in *C. glabrata* (1.58%) [12].

Recently, EUCAST revised the Antifungal Clinical Breakpoint Table with a new version 10.0 valid from 4 Feburary2020 [21]. The CB for micafungin remained unchanged, but an ATU (Area of Technical Uncertainty) comment was added for *C. albicans* isolates with a micafungin MIC of 0.03 mg/L. The new comment states that, if an isolate is susceptible to anidulafungin, it should be reported as susceptible to micafungin and the following comment should be added: “isolates susceptible to anidulafungin with micafungin MIC of 0.03 mg/L do not harbour an *FKS1* mutation conferring resistance to the echinocandins”. If the isolate is not susceptible to anidulafungin, it should be reported as resistant to micafungin and suggest referring to reference laboratory for *FKS1* sequencing and confirmation of MICs [21].

Using the former CB for micafungin of *C. albicans*, a frequent misclassification for borderline resistant strains was noted. Arendrup et al. recently reported ten isolates of *C. albicans* with a micafungin MIC of 0.032 mg/L and an anidulafungin MIC of up to 0.032 mg/L without any target mutations in the hot spot regions [22], thus underlining that the former CB misclassified the genotypical wildtype strains as resistant. We observed the same phenomenon in an even higher number of *C. albicans* isolates (*n* = 86). Still, 23 of our isolates with a MIC of 0.032 mg/L also showed resistance to anidulafungin and they are now classified as resistant to micafungin. According to EUCAST, these isolates should be referred to a reference laboratory for *FKS1* sequencing and the confirmation of MICs and classification. However, none of these isolates showed any mutations in the hot spot regions of *FKS1* and, therefore, are not deemed to be resistant. Thus, the new ATU comment is a major improvement for the correct classification of *FKS1* wildtype isolates. Still, a large part of formerly borderline resistant isolates would have to be sequenced and retested, although *FKS1* mutations seem to be unlikely.

However, missense mutations in the *FKS1* Hot Spot regions are the main cause for echinocandin resistance. This leads to the question of whether these isolates are still part of the wildtype distribution and, consequently, if these isolates are still associated with a therapeutic success. In order to answer these questions, further investigations, e.g., sequencing of the whole *FKS1* gene and other *FKS1* like genes (*GSL1*, *GSL2*), may be beneficial to ensure that no other resistance mutations are present. Another possible investigation could be the measuring of the chitin content of the cell wall. A compensatory overproduction of chitin has also been described in association with echinocandin resistance [23,24]. In the 115 tested isolates, genotypically resistant subpopulations were ruled out. However, we are not able to exclude the phenotypical adaption of a subpopulation [25]. Nevertheless, these mechanisms may only play a minor role.

The fact that no missense mutations were detected within the hotspot regions leads to the conclusion that our isolates, even though they were previously classified as resistant, may not be associated with clinical failure. To answer this question, further in vivo research is inevitable in determining whether micafungin could still be a successful treatment option for *C. albicans* isolates with MICs one or two dilutions above the EUCAST breakpoint. In this study, we raised the question as to whether *C. albicans* isolates with a micafungin MIC of one or two dilutions above the CB of EUCAST are associated with missense mutations in the *FKS1* HS1, HS2, and HS3 regions. No missense mutations within the hotspot regions of the *FKS1* gene in the investigated 115 borderline micafungin resistant isolates could be determined.

## Figures and Tables

**Table 1:** Primer sequences for the polymerase chain reaction.

ID	Hotspot	Overhang- + Specific Locus Sequence	
12F	1, 3	TCGTCGGCAGCGTCAGATGTGTATAAGAGACAGGAACAAGAGATCAAGAAGATATA	Spettel 2019
12R	1, 3	GTCTCGTGGGCTCGGAGATGTGTATAAGAGACAGTGAACGACCAATGGAGAAGA	Spettel 2019
13F	2	TCGTCGGCAGCGTCAGATGTGTATAAGAGACAGCTATGGTCATCCAGGTTTCCA	Garnaud 2015
13R	2	GTCTCGTGGGCTCGGAGATGTGTATAAGAGACAGCACCAACGGTCAAATCAGTG	Garnaud 2015

## Data Availability

The sequencing data is available in the BioProject database under accession number PRJNA695249.
